# The relationship between self-efficacy and uncertainty avoidance levels of school administrators

**DOI:** 10.3389/fpsyg.2025.1554768

**Published:** 2025-04-04

**Authors:** Hamza Öz

**Affiliations:** Faculty of Health Sciences, Yozgat Bozok University, Yozgat, Türkiye

**Keywords:** education management, self-efficacy, school administrators, uncertainty, uncertainty avoidance

## Abstract

Uncertainty and uncertainty avoidance are important factors affecting individuals’ decisions and behaviors. Uncertainty is defined as insufficient information to understand a situation or event or difficulty in coping with conflicting information. The concept of self-efficacy is defined as a person’s belief in their capacity to perform a certain task. This research, which was conducted to determine the relationship between the self-efficacy of school administrators and their levels of uncertainty avoidance, was designed according to the relational screening model in accordance with the quantitative research method. Another aim of the research is to determine whether the relationship between the self-efficacy of school administrators and their levels of uncertainty avoidance varies according to demographic variables. The universe and sample of the research consist of 243 school administrators who were selected from among school administrators working in a province in the Central Anatolian region of Turkey using the simple random sampling method. In the research, the “School Principals’ Self-Efficacy Perception Scale” was used to determine the self-efficacy perceptions of school administrators, and the “Uncertainty Avoidance Questionnaire” was used to determine their levels of uncertainty avoidance. The findings were interpreted in the context of the cultural values of the society in which the research was conducted. The most important results of the research are that the self-efficacy of school administrators is high, their levels of uncertainty avoidance are moderate, and the relationship between the self-efficacy of school administrators and their levels of uncertainty avoidance is low. Various suggestions were made in the context of the limitations of the study.

## 1 Introduction

Uncertainty is a concept that is generally defined as the indecision experienced by individuals when there is not enough information or when the existing information is contradictory (Budner, 1962; Rosen et al., 2007). Accordingly, having sufficient information or not having a contradictory situation may mean that the state of uncertainty can decrease (Korkut-Owen, 2021). If the situation does not contain enough information or the information is contradictory, the individual may experience uncertainty (Budner, 1962). Clampitt and Williams (2004) define uncertainty as an individual approaching a situation with anxiety. The uncertainties that individuals encounter create various behavioral and psychological effects at the personal and social levels. This situation also leads to the emergence of emotional reactions such as stress, anxiety, and restlessness in the interactions of individuals with their environment (Doğan, 2007; Erkenekli, 2013). The individual’s evaluation of uncertainty as threatening, anxiety-provoking, and disturbing leads to a tendency to avoid uncertainty (Buhr and Dugas, 2002; Ladouceur et al., 1998). This tendency manifests itself in situations where individuals experience more anxiety in uncertain environments (Hofstede, 1984; Terzi, 2004).

The tendency to avoid uncertainty is defined as an attitude towards an unknown future, which is related to the individual’s preference for controlling this unknown future or letting it flow (Hofstede, 1984). Cultural differences are also a determining factor in the formation of this tendency. In societies with high levels of uncertainty avoidance, individuals avoid more risks and seek certainty, while in societies with low levels of uncertainty avoidance, uncertainty is accepted as a normal part of life and there is a tendency to take more risks (Hofstede, 1984). It should also be noted that uncertainties are managed not only with formal, explicit rules and laws but also with informal, implicit rules (Hofstede, 1984). In societies with high levels of uncertainty avoidance, employees are observed to prefer to implement the rules determined by managers (Özutku, 2019). Determining the rules to be followed is related to the self-efficacy of managers.

Self-efficacy is the basis of individuals’ beliefs in themselves, and these beliefs have a decisive effect on decisions and behaviors (Bandura, 1995). It is the belief that an individual has the necessary abilities and skills to perform a task (Sharp et al., 2002). A high self-efficacy belief contributes to the formation of high-level performance (Kurt, 2012). In addition, individuals’ self-efficacy perceptions can affect their ability to cope with uncertainty and how they position themselves in social conditions (Bandura, 1986). Ways of coping with uncertainty are directly related to their self-efficacy perceptions. According to Bandura’s self-efficacy theory, a person’s beliefs about performing a task shape their efforts and motivation to accomplish that task (Bandura, 1977). In this context, uncertainty avoidance tendencies and self-efficacy perceptions play an important role in individuals’ decision-making processes and stress-coping mechanisms.

There are many studies in the literature aimed at determining the self-efficacy of school administrators (Açıkgöz, 2022; Bayraktar, 2023; Doğu, 2016; Gökçe, 2014; Gülpınar, 2018; Korkut and Babaoğlan, 2012; Korkut and Keskin, 2015; Köybaşı, 2016; Neyişçi, 2008; Polat and Göktürk, 2005; Tabancali and Celik, 2013). Similarly, there are various studies aimed at determining the levels of uncertainty avoidance of school administrators (Aktaş, 2010; Arbak, 2005; Basım, 2000; Bodur and Kabasakal, 2002; Erdem, 2001; Gürbüz and Bingöl, 2007; Hofstede, 1984; Hofstede, 2001; Köse and Ünal, 2000; Paşa et al., 2001; Sargut, 2015; Saylik, 2017; Sertel et al., 2022; Wasti, 1995; Turan et al., 2005). However, no study has been found examining the relationship between the self-efficacy of school administrators and their levels of uncertainty avoidance.

Ma et al. (2024) suggest that the regulatory role of uncertainty avoidance at the group and organizational level should be investigated. Watts et al. (2020) emphasize that Hofstede’s cultural values should be examined in individual, organizational, and regional contexts and their relationships with other variables should be examined. In addition, Baltacı (2017) conducted a study aiming to determine the relationship between school principals’ self-efficacy perceptions and professional attitudes and stated that there is a need for studies examining the self-efficacy perceptions of school principals.

While self-efficacy is considered an important factor in the context of learning and teaching in the study conducted by Huang et al. (2024), the research conducted by García-Alvarez et al. (2024) emphasizes that leaders with high self-efficacy levels play a mediating role in strengthening their self-efficacy and the importance of studies on determining the self-efficacy of leaders. This study will not only fill this gap in the literature but will also contribute to determining the relationship between school administrators’ self-efficacy perceptions and uncertainty avoidance levels.

## 2 Literature review

### 2.1 Uncertainty avoidance

Uncertainty is a situation that triggers anxiety and uncertainty avoidance behaviors because individuals cannot fully define and make sense of the situation (Teoh and Foo, 1997; Yeloğlu, 2011). Uncertainty avoidance is considered the most remarkable concept among all cultural dimensions because it expresses how uncomfortable people are in the face of unknown or uncertain things (Hofstede, 1984, 2001). This cultural tendency reflects the factors that determine how uncomfortable or comfortable individuals in a society can be in new, unfamiliar, and unusual conditions (Zhou et al., 2022). Ultimately, uncertainty is a situation related to the individual’s search for the truth (Demirel and Amanor, 2023). In this context, not being able to cope with uncertainty usually leads to uncertainty avoidance tendencies in individuals.

Uncertainty avoidance refers to the level of anxiety that individuals feel in environments where they have insufficient or contradictory information and where there are complex and rapid changes (Hofstede, 1984). Terzi (2004) defines this situation as the level of anxiety experienced due to a lack of information, unpredictable changes, and complex conditions. Korkut and Keskin (2015) see uncertainty avoidance as the tendency of individuals to cope with situations such as indecision, disorder, and unpredictability caused by lack of information and emphasize that this can affect individuals’ decisions and lives.

Uncertainty avoidance relates to how threatening a culture perceives uncertainty and change. Societies with low uncertainty avoidance are more adaptable and open to new ideas, while societies with high uncertainty avoidance often have rigid norms, formal systems, and an aversion to change. Cultures with high uncertainty avoidance tend to prefer consistency, predictability, and clear-cut regulations (Dursun, 2013; Yeşil, 2012). In these cultures, elements such as job stability, clear expectations, and controlled work environments may have an impact on job satisfaction. Conversely, societies that encourage flexibility, adaptability, and entrepreneurial spirit may have low levels of uncertainty avoidance. Autonomy, creativity, and the chance to take risks may have an impact on a person’s job satisfaction in these environments. Cultures that tolerate uncertainty well and reject uncertainty less often may be more adaptable and tolerant of change. They may be open to trying new things and taking risks; This can have an impact on employees in dynamic, rapidly changing organizations that require creativity and agility (Demirel and Amanor, 2023; Hofstede, 1984).

Uncertainty avoidance culture aims to reduce such uncertain conditions by establishing strict laws and standards in societies. However, societies with high uncertainty avoidance may have a more emotional structure and intrinsic motivational sources may be more prominent (Boubakri et al., 2021; Tang and Zhou, 2022). Individuals experiencing uncertainty may feel threatened and may have emotional reactions such as restlessness, anxiety, and worry (Erkenekli, 2013; Doğan, 2007). In addition, uncertainty avoidance is a condition that determines how much people can tolerate future uncertainties (Birsel et al., 2009).

Individuals who encounter uncertainty and try to overcome this situation by seeking definitive answers have low uncertainty tolerance (Wang, 2018). Situations where the risks associated with uncertainty are high or the outcomes are uncertain create a stronger desire to avoid uncertainty in individuals (Aydın and Uçman, 2019). In societies where uncertainty avoidance is high, there is more anxiety about the future and a tendency to avoid risk. Societies with low uncertainty avoidance prefer to take more risks and accept innovations (Terzi, 2004).

Uncertainty avoidance is related to the tolerance levels of societies towards uncertain and complex events (Azizoğlu, 2011), and it arises from the cultural differences of societies, economic conditions, social structure, and social factors (Çaloğlu, 2014). In societies with low uncertainty avoidance levels, individuals can tolerate uncertainty more easily, while in societies with high uncertainty avoidance levels, risk avoidance, and control-seeking are more dominant (Hofstede, 2001). For example, societies with low uncertainty avoidance levels such as England, Denmark, Sweden, and Ireland accept uncertainty as a normal part of life, while societies such as Japan, Portugal, Greece, and Spain tend to avoid risk (Hofstede, 1984). Studies show that Turkey is among the countries with high uncertainty avoidance and low tolerance for uncertainty (Hofstede, 1984; Sığrı and Tığlı, 2006; Şekerli and Gerede, 2011).

### 2.2 Self-efficacy

The basic point of the concept of self-efficacy is that individuals are more likely to perform actions for which they feel competent and less likely to perform actions for which they feel incompetent. When a person successfully performs a task, it reinforces their belief that they can successfully perform such tasks in the future. In this respect, the beliefs that individuals develop about their abilities play an important role in determining what they can achieve. Bandura (1977) defines self-efficacy as a person’s belief in their abilities in terms of planning and implementing the courses of action needed to achieve future goals. Sharp et al. (2002) sees this as the most important predictor of individual behavior; Lunenburg (2011) defines self-efficacy as an action-specific version of an individual’s self-esteem.

Bandura (1989) examined the basic characteristics underlying self-efficacy under the titles of cognitive processes, emotional processes, and control processes. Cognitive processes are related to goal setting. Individuals with high self-efficacy tend to set larger goals, undertake more challenging tasks, and exert more effort to achieve their goals. These individuals tend to imagine successful outcomes rather than negative outcomes. Emotional processes are related to belief status; a person’s belief in their abilities affects not only their motivation but also the severity of stress and depression experienced when coping with risky and difficult situations. Emotional reactions can change the thought process and directly or indirectly affect actions. In addition, these reactions depend on people’s beliefs in their coping capacity. Individuals who believe they can cope with risks are less disturbed by these risks and can reduce their stress by controlling the risks (Bandura, 1995). The control process is related to internal control and refers to the individual’s perception of the fundamental causes of the events they experience. People may believe that their experiences are controlled by external forces such as fate or luck, or by internal forces such as personal decisions and efforts (Arseven, 2016).

Self-efficacy focuses on a person’s beliefs about whether they can perform a certain task. A person with high self-efficacy believes that success requires the use of cognitive and emotional processes. This is an example of a belief in the existence of an internal control process. An individual’s perception of self-efficacy is shaped by information obtained from four primary sources: direct experiences, indirect experiences, verbal persuasion, and psychological states. A person’s direct experiences of positive or negative experiences support their self-efficacy beliefs regarding similar situations in the future (Cattelino et al., 2019). What is learned through observation also contributes indirectly to their self-efficacy beliefs. While verbal persuasion refers to encouragement and advice given to an individual about whether they can succeed in an activity, psychological states focus on how an individual’s stress or anxiety levels affect their perception of self-efficacy. A psychologically comfortable individual has a higher self-efficacy expectation that they will complete a task. In this case, positive mood strengthens self-efficacy beliefs, while negative emotions such as depression and hopelessness weaken this belief (Bandura, 1982; Pajaras, 2009; Tepe, 2011).

According to Bandura (1977), self-efficacy is situation-specific and cannot be determined in general, while Zimmerman (2000) states that self-efficacy belief is multidimensional and is related to different areas. Studies have shown that individuals’ self-efficacy beliefs affect their behaviors (Bandura, 1977; Enochs and Riggs, 1990). Therefore, questioning self-efficacy beliefs is quite useful in explaining and understanding individual behaviors. Self-efficacy perception enables individuals to make the most appropriate choice in various situations, to maintain their efforts in the face of obstacles, and to exhibit productive behaviors instead of emotional reactions (Bandura, 1986). Therefore, educators’ self-efficacy is critical for effective teaching. It is defined as a set of beliefs regarding a teacher’s ability and capacity to educate and influence students’ behaviors and goals, regardless of external influences or obstacles (Bandura, 1997; Tschannen-Moran and Hoy, 2001).

According to García-Alvarez et al. (2024), they revealed that leadership positively affects self-efficacy. In addition, general self-efficacy strongly affects academic self-efficacy. School administrators with high self-efficacy beliefs can plan and organize effective teaching, set specific, achievable goals, and have high expectations (Sharma and George, 2016; Zee and Koomen, 2016). They establish positive relationships with their immediate work environment (colleagues, school principal, parents), thus contributing to the promotion of learning through a positive communication framework for the school as a whole. On the other hand, administrators with a low sense of self-efficacy are pessimistic, have low self-esteem, experience stress, cannot fulfill teaching tasks, are less organized and systematic, are rigid, critical, and exert external control over the classroom (Tschannen-Moran and Hoy, 2001).

## 3 Methods

### 3.1 Purpose of research

The main purpose of the research is to determine the relationship between the self-efficacy of school administrators and their uncertainty avoidance levels. In this context, answers to the following questions will be sought to achieve the purpose of the research.

1. What is the level of self-efficacy and uncertainty avoidance of school administrators?
2. Do the self-efficacy and uncertainty avoidance levels of school administrators differ according to gender, marital status, age, and seniority?
3. What is the relationship between the self-efficacy and uncertainty avoidance levels of school administrators?
4. Can the uncertainty avoidance levels of school administrators be predicted through their self-efficacy?

### 3.2 Research model

This study, which was conducted to determine the relationship between the self-efficacy of school administrators and their uncertainty avoidance levels, was designed according to the relational screening model, one of the quantitative research methods. Relational screening model, the relational screening model is a screening model that examines the relational change between two or more variables. The relational screening model tries to find out whether the variables change together or not, and if a change is observed, how (Karasar, 2017). This study was carried out to measure the relationship between the self-efficacy of school administrators and their uncertainty avoidance levels according to the perceptions of the school administrators participating in the research. In this context, correlational design and intergroup comparison design were used. In the correlational design, the relationships between the concepts were examined with correlation analysis and regression analysis. In addition, in the intergroup comparison design, the effects of the parameters thought to affect the variables were examined with directed comparison analyzes (Karasar, 2017).

### 3.3 Universe and sample

The universe of the study consists of 648 school administrators working in public schools affiliated with the Yozgat Provincial Directorate of National Education. The sample of the study consists of 243 school administrators determined using the simple random sampling method. Since it was not possible to reach all of the school administrators in the research universe, a sample was taken from the universe. The simple random sampling method was used during the creation of the sample of the study and the probability of selection of each individual in the universe was evaluated equally, thus allowing individuals to have the same opportunity. In other words, in this method, everyone has an equal chance of being selected and the preferences of individuals do not affect others (Büyüköztürk, 2020). When determining the sample size, a 95% confidence level and a 5% margin of error were taken as basis, and in this context, data were collected on the basis that a sample of 243 people could represent the universe of 648 people (Balcı, 2018). Descriptive information about the sample of the study is presented in Table 1.

**Table 1:** Descriptive information of participants.

Variables	Category	f	%
Gender	Woman	36	14.8
	Male	207	85.2
Marital Status	Single	42	17.3
	Married	201	82.7
Age	Between 26 and 35 years old	34	14.0
	Between 36 and 45 years old	81	33.3
	Between 46 and 55 years old	91	37.4
	Ages 56 and over	37	15.3
Seniority	0–10 years	31	12.7
	Between 11 and 15 years	30	12.4
	Between 16 and 20 years	63	25.9
	Between 21 and 25 years	62	25.5
	26 years and above	57	23.5

### 3.4 Data collection tools

*“Uncertainty Avoidance Questionnaire*” developed by Korkut and Keskin (2015), for which the necessary permissions were obtained, and the “*School Principals’ Self-Efficacy Perception Scale”* developed by Tschannen-Moran and Hoy (2001) and adapted into Turkish by Baltacı (2017) were used as research data collection tools. These scales were used in the literature review because they were believed to be sufficient scales to determine the uncertainty avoidance and self-efficacy perception levels of school administrators.

The school principals’ self-efficacy perception scale, developed by Tschannen-Moran and Hoy (2001) and adapted into Turkish by Baltacı (2017), consists of three dimensions (administrative, instructional, and ethical/moral) and 18 items. The scale has a five-point Likert-type rating ranging *from (1) Extremely Inadequate to (5) Extremely Adequate.* The values obtained by the five-point rating scale used are classified as *Never (1.00–1.79), Rarely (1.80–2.59), Sometimes (2.60–3.39), Often (3.40–4.19) and Always (4.20–5.00). In addition, in the analysis regarding the item total correlations of the scale, it was determined that all items had values over 0.40.* Cronbach’s Alpha reliability coefficient of the scale was 0.88; The composite reliability coefficient was determined as 0.92.

The uncertainty avoidance questionnaire consists of a total of 16 items including opinions on uncertainty avoidance developed by Korkut and Keskin (2015). A five-point Likert-type rating scale consisting of *(5) Totally Agree, (4) Very Agree, (3) Moderately Agree, (2) Slightly Agree and (1) Strongly Disagree* options was used in the data collection tool; two items were reverse scored. The mean scores obtained by the five-point rating scale used were divided into equal parts between 1 and 5 and the obtained values were classified as *Totally Disagree (1.00–1.79), Slightly Agree (1.80–2.59), Moderately Agree (2.60–3.39), Very Agree (3.40–4.19) and Strongly Agree (4.20–5.00).*

### 3.5 Data collection and analysis

The research began with a comprehensive literature review. The variables of the study were determined based on literature findings. Necessary permissions were obtained for the implementation of the data collection tools. A personal information form developed by the researchers was added to the data collection tool. Within the scope of the research, 563 school administrators out of the 648 school administrators that constituted the study universe were reached, but only 243 school administrators responded. Data were collected through a form created in an electronic environment.

The collected data were analyzed with the help of pairwise and multiple comparison techniques via the SPSS package program. The distribution properties of the data sets were examined before the analysis. It was investigated whether the data showed normal distribution according to the variables to be compared. Kolmogorov- Smirnov test and skewness and kurtosis values were used to determine the distribution properties of the data sets and since the skewness and kurtosis values were between +1.5 and − 1.5, it was seen that the data showed normal distribution. Since it was determined that the distribution of the data showed normal distribution according to our dependent and independent variables, it was decided to use parametric tests during the data analysis phase. To perform a parametric test, 30 is considered sufficient as the number that should be in a group (Büyüköztürk, 2020; Karasar, 2017). Especially in this study, although there is a great difference between the groups in the gender and marital status variables, parametric analysis was performed because the data showed a normal distribution and a sufficient number was reached to perform parametric analysis. It was also checked whether there was missing data in the data set, and it was seen that there were no missing values. Then, it was checked whether the extreme values disrupted the normal distribution, and it was determined that there were no extreme values in the data sets.

To test the reliability of the scales used in data collection, Cronbach’s Alpha value was examined. This value of the school principals’ self-efficacy perception scale was found to be 0.96 and it was determined to be highly reliable. Cronbach’s Alpha value of the uncertainty avoidance questionnaire was found to be 0.91 and it was decided that the research was highly reliable in both scales used in the research. In the research, the perceived self-efficacy and uncertainty avoidance levels of school principals working in schools were determined according to the variables of the unit, gender, marital status, age, and seniority of the participants, and the relationships between the variables were tried to be revealed. The self-efficacy and uncertainty avoidance levels of the school principals included in the scope of the research were determined in terms of arithmetic mean and standard deviation. The means of the variables of the unit, gender, marital status, age, and seniority of the participants were determined. In the test of the differences between the means, the t-test was preferred for paired groups in parametric distributions, and the ANOVA test was preferred for multiple groups. The correlational relationship between self-efficacy and uncertainty avoidance was determined by Pearson Product Moment Correlation Analysis and regression analysis was conducted to determine the predictive level.

## 4 Results

In this section, the findings obtained in the context of the research questions are given. The findings are presented and interpreted in tables.

### 4.1 Findings regarding school administrators’ self-efficacy and uncertainty avoidance levels

The arithmetic means and standard deviation values of school administrators’ self-efficacy and uncertainty avoidance levels are presented in Table 2.

**Table 2:** Values relating to self-efficacy and uncertainty avoidance levels.

Scales and sub-dimensions	n	X̅	SS	Minimum	Maximum
Self-Efficacy	243	4.39	0.02	3.61	5.00
Administrative dimension	243	4.43	0.02	3.50	5.00
Educational dimension	243	4.29	0.03	3.00	5.00
Ethical/moral dimension	243	4.44	0.03	2.50	5.00
Uncertainty avoidance	243	2.97	0.02	2.06	3.56

Looking at Table 2, it is seen that the arithmetic means of the self-efficacy levels of school administrators are in total (X̅=4.39), administrative dimension (X̅=4.43), instructional dimension (X̅=4.29) and ethical/moral dimension (X̅=4.44). According to these means, it can be stated that the self- efficacy of school administrators has a high average value at the level of “always*.”* It is seen that the level of uncertainty avoidance of school administrators has a medium value with the arithmetic mean (X̅= 2.97). According to these values, it can be found that the self-efficacy of school administrators is high, and their level of uncertainty avoidance is medium. It is important that school administrators with high self-efficacy also have a moderate level of uncertainty avoidance. This can be considered an important finding in terms of the relationship between school administrators’ self-efficacy and uncertainty avoidance levels. A high level of self-efficacy and a moderate level of uncertainty avoidance can also be interpreted as an increase in self-efficacy leading to avoidance of uncertainty.

### 4.2 Findings regarding the comparison of self-efficacy and uncertainty avoidance levels of school administrators in terms of variables

The *t*-test results for comparing the self-efficacy of school administrators in terms of demographic variables are presented in Table 3.

**Table 3:** *T*-test results for comparing self-efficacy of school administrators in terms of demographic variables.

Variables	Category	n	General		Administrative		Instructional		Ethics/Moral	
			X̅ / SS	t p	X̅ / SS	t p	X̅ / SS	t p	X̅ / SS	t p
Gender	Woman	36	4.32/0.47	1,049	4.40/0.38	0.497	4.11/0.69	2,415	4.47/0.43	0.301
	Male	207	4.40/0.38	0.36	4.43/0.40	0.62	4.33/0.46	0.07	4.44/0.48	0.76
Marital status	Single	42	4.43/0.40	0.325	4.50/0.44	0.589	4.33/0.55	0.249	4.45/0.33	0.062
	Married	201	4.39/0.40	0.74	4.43/0.39	0.55	4.29/0.50	0.80	4.44/0.48	0.95

Looking at Table 3, it is seen that the self-efficacy levels of school administrators do not differ according to gender and marital status variables. This situation reveals the finding that the perception of school administrators does not differ in terms of self-efficacy level according to gender and marital status variables.

The *t*-test results for comparing the uncertainty avoidance levels of school administrators in terms of demographic variables are presented in Table 4.

**Table 4:** *T*-test results for comparing the uncertainty avoidance levels of school administrators in terms of demographic variables.

Variables	Category	n	Uncertainty Avoidance General	
			X̅ / SS	t p
Gender	Woman	36	2.91/0.25	2,166
	Male	207	2.77/0.32	0.00 *
Marital status	Single	42	2.95/0.33	1,735
	Married	201	2.79/0.31	0.08

Analyzing Table 4, it is seen that the uncertainty avoidance levels of school administrators differ significantly (*p* = 0.00) according to the gender variable. When evaluated according to the arithmetic means as a result of the analysis, it was found that this difference was higher in women (X̅=2.91). In other words, the uncertainty avoidance level of school administrators is higher in female school administrators. From the analysis in Table 4, it is seen that the uncertainty avoidance levels of school administrators do not differ according to the marital status variable (*p* = 0.08).

The results of the ANOVA test to compare the self-efficacy of school administrators in terms of demographic variables are presented in Table 5.

**Table 5:** ANOVA test results for comparing self-efficacy of school administrators in terms of demographic variables.

Variables	Category	n	General		Administrative		Instructional		Ethics/Moral	
			X̅/SS	F p Diff.	X̅/SS	F p Diff.	X̅/SS	F p Diff.	X̅/SS	F p Diff.
Age	Between 26 and 35 years old	34	4.18/0.45	5.343	4.31/0.45	1.609	3.95/0.64	7.879	4.27/0.44	3.641
	Between 36 and 45 years old	81	4.35/0.42	0.01	4.40/0.42	0.18	4.23/0.54	0.00	4.41/0.54	0.01
	Between 46 and 55 years old	91	4.50/0.37	1–3	4.49/0.38		4.46/0.41	1–3	4.56/0.45	1–3
	Ages 56 and over	37	4.36/0.17		4.46/0.29		4.27/0.26	2–3	4.35/0.14	
Seniority	0–10 years	31	4.26/0.42	6.163	4.40/0.40	2.755	4.00/0.67	6.576	4.38/0.35	6.710
	Between 11 and 15 years	30	4.10/0.50	0.00	4.25/0.43	0.02	3.98/0.55	0.00	4.06/0.75	0.00
	Between 16 and 20 years	63	4.44/0.35	2–3	4.46/0.38	2–5	4.36/0.48	2–3	4.50/0.37	2–3
	Between 21 and 25 years	62	4.44/0.41	2–4	4.40/0.44		4.36/0.48	2–4	4.56/0.44	2–4
	26 years and above	57	4.47/0.28	2–5	4.53/0.29		4.41/0.34	2–5	4.47/0.37	2–5

Analyzing Table 5, it is seen that the self-efficacy levels of school administrators did not differ according to the age variable in the administrative dimension (*p* = 0.18), but they differed significantly in the instructional dimension (*p* = 0.00), ethical/moral dimension (*p* = 0.01) and general self-efficacy dimensions (*p* = 0.01). According to the results of the Tukey post-hoc analysis conducted to determine which categories this differentiation occurred between, it was found that in the instructional dimension, the participants in the 26–35 age range (X̅=3.95) and the participants in the 36–45 age range (X̅=4.23) were lower than the participants in the 46–55 age range (X̅=4.46). In the ethical/moral dimension, the participants in the 26–35 age range (X̅ =4.27) were lower than the participants in the 46–55 age range (X̅ =4.56). Similarly, it is seen that the general self-efficacy level of the participants in the 26–35 age range (X̅=4.18) is lower than the participants in the 46–55 age range (X̅=4.50).

From the analysis in Table 5, the self-efficacy levels of school administrators differ significantly in all dimensions according to the seniority variable. According to the results of the Tukey post-hoc analysis conducted to determine between which categories this differentiation occurred, in the general self-efficacy dimension, participants with 11–15 years of seniority (X̅=4.10) were at a lower level than participants with 16–20 years of seniority (X̅=4.44), participants with 21–25 years of seniority (X̅=4.44) and participants with more than 26 years of seniority (X̅=4.47); in the administrative dimension, participants with 11–15 years of seniority (X̅=4.25) were at a lower level than participants with 26 years of seniority and more (X̅=4.53); in the instructional dimension, participants with 11–25 years of seniority (X̅=3.98) were at a lower level than participants with 16–20 years of seniority (X̅=4.36), 21–25 years of seniority (X̅=4.47). (X̅=4.36) and 26 years and above seniority (X̅=4.41) are lower than the participants and similarly, in the ethical/moral dimension, the participants with 11–25 years of seniority (X̅=4.06) are lower than the participants with 16–20 years of seniority (X̅=4. 50), 21–25 years of seniority (X̅=4.56) and 26 years and above seniority (X̅=4.47).

The results of the ANOVA test to compare the uncertainty avoidance levels of school administrators in terms of demographic variables are presented in Table 6.

**Table 6:** ANOVA test results for comparing the uncertainty avoidance levels of school administrators in terms of demographic variables.

Variables	Category	n	Uncertainty Avoidance	
			X̅/SS	F p Difference
Age	Between 26 and 35 years old	34	2.89/0.28	1.523
	Between 36 and 45 years old	81	2.78/0.32	0.20
	Between 46 and 55 years old	91	2.81/0.28	
	Ages 56 and over	37	2.70/0.40	
Seniority	0–10 years	31	2.85/0.29	4.385
	Between 11 and 15 years	30	2.85/0.27	0.00
	Between 16 and 20 years	63	2.77/0.31	5–4
	Between 21 and25 years	62	2.88/0.29	
	26 years and above	57	2.67/0.32	

Looking at Table 6, it is seen that the uncertainty avoidance levels of school administrators do not differ significantly according to the age variable (*p* = 0. 20) but differ significantly according to the seniority variable (*p* = 0.00). Tukey post-hoc analysis was conducted to determine between which categories this difference occurred according to the seniority variable. As a result of this analysis; It was found that the uncertainty avoidance levels of participants with 26 years of seniority and above (X̅=2.67) were lower than those with 21–25 years of seniority (X̅=2.88).

### 4.3 Findings regarding the relationship between self-efficacy and uncertainty avoidance levels of school administrators

Pearson Product Moment Correlation Analysis was conducted since our data showed a normal distribution and the analysis results are presented in Table 7.

**Table 7:** Results of Pearson product moment correlation analysis conducted to determine the relationship between self-efficacy and uncertainty avoidance levels of school administrators.

Points	1	2	3	4	5
1.Self-efficacy	1	0.841 **	0.902 **	0.861 **	−0.235 **
2. Administrative	0.841 **	1	0.671 **	0.571 **	−0.145 *
3. Educational	0.902 **	0.671 **	1	0.654 **	−0.220 **
4. Ethics/Morality	0.861 **	0.571 **	0.654 **	1	−0.237 **
5. Uncertainty Avoidance	−0.235 **	−0.145 *	−0.220 **	−0.237 **	1

*N* = 243, ^*^p < 0.05, ^**^p < 0.01.

Table 7 presents the relationship between school administrators’ self-efficacy and uncertainty avoidance levels. Accordingly, while there is a high level of relationship between self-efficacy and its sub-dimensions, the relationship between general self-efficacy level and uncertainty avoidance levels is negatively low (*p* = −0. 235), similarly, the relationship between administrative dimension and uncertainty avoidance level is negatively low (*p* = −0.145), the relationship between instructional dimension and uncertainty avoidance is negatively low (*p* = −0.220), and this relationship is negatively low (*p* = −0.237) in the ethical/moral dimension. This finding reveals the result that the relationship between school administrators’ self-efficacy and uncertainty avoidance levels is low.

### 4.4 Findings regarding the prediction levels of school administrators’ self-efficacy on uncertainty avoidance

To determine to what extent, the self-efficacy of school administrators predicts their levels of uncertainty avoidance, it was checked whether the prerequisites of regression analysis (normality, linearity, correlation, and other factors) were met and when it was seen that the prerequisites were met, multiple linear regression analysis was performed, and the analysis results are presented in Table 8.

**Table 8:** Multiple linear regression analysis results showing the prediction level of school administrators’ self-efficacy on uncertainty avoidance levels.

Predictor Variables		Predicted Variable Uncertainty Avoidance
		[R = 0.254; R2 = 0.065] *F* = 5.496 p = 0.00
Constant	t	15,218
	p	0.00
Administrative	β	0.036
	t	0.532
	p	0.59
Instructional	β	−0.086
	t	−1,469
	p	0.14
Ethics/Moral	β	−0.114
	t	−2,026
	p	0.04

^*^p < 0.05, ^**^p < 0.01.

Looking at Table 8, the dimensions of self-efficacy of school administrators in the model, namely administrative, instructional, and ethical/moral variables, have a low level and significant relationship with uncertainty avoidance levels (*R* = 0.254; *p* < 0.01). The dimensions of self-efficacy of school administrators explain approximately 0.7% of the total variance of uncertainty avoidance level (R^2^ = 0.065). The relative order of importance of predictor variables on uncertainty avoidance is the administrative dimension (*β* = −0.036), instructional dimension (*β* = −0.086), and ethical/moral dimension (*β* = −0.114). According to the t-test results, it is the administrative dimension (*t* = 0.532), instructional dimension (*t* = −1.469), and ethical/moral dimension (*t* = −2.026). The results of this analysis show that the self-efficacy of school administrators predicts the levels of uncertainty avoidance, albeit at a low level, that is, the uncertainty avoidance levels of school administrators can be explained by a very low level of relationship of 0.7% over their self-efficacy levels.

## 5 Discussion

The fact that the self-efficacy levels of school administrators are “always” high in all dimensions is among the most important findings of this study. Similar to this finding, it is possible to come across studies in the literature that find the self-efficacy perceptions of school administrators to be high (Açıkgöz, 2022; Arıkan, 2019; Bayraktar, 2023; Doğu, 2016; Gülpınar, 2018; Köybaşı, 2016; Tabancali and Celik, 2013). This important finding can be explained by the fact that school administrators have a high level of belief in their academic and administrative skills in managing their schools when self-efficacy is defined as a person’s belief that they can do a job (Arseven, 2016; Bandura, 1997; Karataş and Başbay, 2014).

In this study, it was found that the self-efficacy levels of school administrators did not differ according to some demographic variables but differed according to some variables. The finding that the study did not differ according to the gender variable is consistent with the studies conducted by Bayraktar (2023) and Açıkgöz (2022). However, the finding that it did not differ according to the marital status variable is not consistent with the research conducted by Açıkgöz (2022). In the study of Açıkgöz (2022), it was concluded that married school administrators see themselves as more competent than single school administrators. According to the age variable, differentiation was observed in some dimensions of self-efficacy, and this finding is also found in similar studies in the literature (Açıkgöz, 2022). According to the seniority variable, it was found that the level of self-efficacy increases as the length of seniority increases, and this finding is consistent with the finding that there is a significant relationship between seniority and self-efficacy in the studies conducted by Williams (2012) and Negis-Işık and Derinbay (2015). The finding of this study regarding the seniority variable is also not consistent with the finding that the self-efficacy levels of school administrators did not differ according to the seniority variable in the studies conducted by Bayraktar (2023) and Açıkgöz (2022).

Although there are studies in the literature that find the level of avoiding uncertainty of school administrators to be high (Aktaş, 2010; Arbak, 2005; Basım, 2000; Bodur and Kabasakal, 2002; Erdem, 2001; Gürbüz and Bingöl, 2007; Hofstede, 1984; Hofstede, 2001; Köse and Ünal, 2000; Paşa et al., 2001; Sargut, 2015; Saylik, 2017; Wasti, 1995; Turan et al., 2005), it is also possible to come across studies that find it to be at a moderate level, which is consistent with the findings of this study (Korkut and Keskin, 2015; Neyişçi, 2008; Polat and Göktürk, 2005). In the study conducted by Gökçe (2014), it was determined that the level of uncertainty avoidance of school administrators is low. In societies where the level of uncertainty avoidance is low, differences are not seen as a threat and the level of anxiety is low, while in societies where uncertainty avoidance is high, such as Turkey, there is a focus on career goals, an inability to go beyond the ordinary, and intolerance to differences (Han and Saylık, 2021; Hofstede, 2011; Hofstede et al., 2010). It is important that the level of uncertainty avoidance of school administrators in this study is moderate. It is important that the level of uncertainty avoidance of school administrators in this study was found to be at a moderate level, as the lower the level of uncertainty avoidance, the more flexible working and risk-taking opportunities will be opened (Su et al., 2022).

In this study, it was concluded that the levels of school administrators’ uncertainty avoidance did not differ according to the variable of marital status. It is possible to come across studies in the literature that are consistent with this finding (Karşu Cesur, 2015; Saylik, 2017;). In the study conducted by Yörük (2009), it was found that the level of uncertainty avoidance differed according to the marital status variable. Yörük (2009) reveals that the levels of uncertainty avoidance of married participants are higher than single participants. Yörük (2009) attributes this finding to the responsibility and indirect anxiety level of married participants brought by being a family, and to the fact that single people are only responsible for themselves and are relatively freer and less anxious. It is also possible to come across studies supporting the finding that the levels of uncertainty avoidance of school administrators do not differ according to age variables (Dalğali, 2020; Neyişçi, 2008; Turan et al., 2005).

In this study, the finding that the levels of uncertainty avoidance of school administrators differ significantly according to the gender variable and that women have a higher level of uncertainty avoidance than men is explained by the fact that women are considered to be among the disadvantaged groups in Turkish society, a situation closely related to the cultural values of the society. Similarly, Korkut and Keskin's (2015) study found that women have a lower tolerance for uncertainty compared to men. However, Dalgün (2011) revealed in his study that men have a higher level of uncertainty avoidance than women. However, in the study conducted by Saylik (2017), it was found that the levels of school administrators’ uncertainty avoidance did not differ significantly according to the gender variable. Similarly, in the study conducted by Turan et al. (2005), it was found that the levels of administrators’ uncertainty avoidance did not differ according to the gender variable.

In this study, it was determined that the levels of school administrators’ uncertainty avoidance differed significantly according to the seniority variable. In the study conducted by Neyişçi (2008), it was found that school administrators with high seniority tended to avoid uncertainty more. Dalgün (2011) reached a conclusion in his study that the uncertainty avoidance levels of employees with less than 1 year and more than 14 years were higher than other employees. In the study conducted by Turan et al. (2005), it was found that the uncertainty avoidance levels of administrators did not differ according to the seniority variable.

In this study, it was determined that there was a high-level relationship between the self-efficacy of school administrators and their levels of uncertainty avoidance and that there was a low-level relationship in the opposite direction between the self-efficacy level and uncertainty avoidance levels. Similarly, in the study conducted by Dalğali (2020), a low-level relationship was found between leadership behaviors and uncertainty avoidance. In the study conducted by Turan et al. (2005), it was concluded that administrators preferred situations determined by rules to uncertain situations. Rhodes (2012) defines self-efficacy as an important indicator of people’s abilities and potential. Accordingly, it seems possible for school administrators with high self-efficacy to reveal their potential in managing conflict situations that arise. In fact, in the study conducted by İnandı et al. (2013), it was found that the perception of self-efficacy has a strong relationship with conflict resolution strategies. People with high uncertainty avoidance tend to avoid loss of resources, including support from leaders (Ma et al., 2024; Sartono et al., 2024). Especially in educational institutions, the presence of school administrators with high self-efficacy is considered important in resolving conflict situations that arise due to uncertainty.

In this study, it was found that the self-efficacy of school administrators predicted uncertainty avoidance levels, albeit at a low level, that is, the uncertainty avoidance levels of school administrators could be explained by a very low level of relationship of 0.7% over their self-efficacy levels. The low level of self-efficacy explaining the uncertainty avoidance levels of school administrators (0.7%) can be explained by the fact that other variables such as personality traits, organizational support, norms, and cultural values are effective on uncertainty avoidance behavior (Pham and Do, 2024). Ayoun and Moreo (2008) comparatively examined the relationship between the strategic decision-making processes of administrators and the uncertainty avoidance dimension in their study and could not find a significant relationship between them in terms of the variables examined. Bauman (2006) concluded that the leadership practices of administrators were predicted by self-management at the highest level.

## 6 Conclusion

In this study, which was conducted to determine the relationship between the self-efficacy of school administrators and their levels of uncertainty avoidance, it was concluded that the self-efficacy of school administrators had a high level of “always” in all dimensions and their levels of uncertainty avoidance had a “medium” level of value.

In this study, it was found that the self-efficacy of school administrators did not differ significantly according to gender and marital status variables but differed according to age and seniority variables. The result is that the self-efficacy perceptions of school administrators who are older are especially important. In this study, it was also found that the levels of school administrators’ uncertainty avoidance did not differ according to marital status and age variables but differed according to seniority and gender variables. The fact that women have a higher level of uncertainty avoidance than men in terms of uncertainty avoidance can be explained by the fact that women are considered to be in disadvantaged groups in Turkish society and this is closely related to the cultural values of the society. According to the seniority variable, it is important that individuals with higher seniority have a lower level of uncertainty avoidance perception.

One of the most important results of this research is that there is a high level of relationship between self-efficacy and uncertainty avoidance levels of school administrators and their self-efficacy and its sub-dimensions, and a low level of relationship in the opposite direction between self-efficacy and uncertainty avoidance levels. This result shows that there is a reverse relationship between self-efficacy and uncertainty avoidance. In other words, school administrators with high self-efficacy have low uncertainty avoidance levels. Individuals with low uncertainty avoidance levels will need less support from school administrators. It has been determined that school administrators’ self-efficacy predicts uncertainty avoidance levels, albeit at a low level, that is, school administrators’ uncertainty avoidance levels can be explained with a very low level of relationship of 0.7% over their self-efficacy levels. The fact that school administrators’ self-efficacy explains their uncertainty avoidance levels at a low level (0.7%) is explained by the fact that other variables such as personality traits, organizational support, norms, and cultural values are effective on uncertainty avoidance behavior.

### 6.1 Limitations and recommendations

The fact that this research was conducted in a province located in the central Anatolian region of Turkey with only school administrators can be considered as a limitation of the research. School administrators in other regions or countries with different cultural contexts may exhibit different self-efficacy and uncertainty avoidance patterns. In this context, it is recommended for future studies to repeat the study according to the perceptions of school administrators and/or teachers working in different provinces or different countries and to determine how they differ. Another limitation is that this study was conducted with a limited sample group (243) using the quantitative research method. Therefore, the study can be repeated with a larger sample group selected from different regions using qualitative and/or mixed methods. In addition, this study can be repeated with longitudinal studies in an experimental sense in order to determine how self-efficacy and uncertainty avoidance levels evolve over time.

In order to reduce uncertainty avoidance and increase self-efficacy levels, professional development programs, workshops, and in-service training activities can be recommended. Since increasing the studies conducted to increase the self-efficacy of school administrators will contribute to the reduction of uncertainty situations, it can be recommended that these studies be made into public policy. In addition, it may be recommended to include studies that can reveal the effects of personality traits, organizational support and cultural values that are thought to have an effect on uncertainty avoidance behavior.

## Data Availability

The original contributions presented in the study are included in the article/supplementary material, further inquiries can be directed to the corresponding author.
